# High ventilation breathwork as a non-pharmacological model for inducing psychedelic-like states: expert evaluations of therapeutic potential and risk

**DOI:** 10.1038/s44184-026-00246-x

**Published:** 2026-09-17

**Authors:** Ulrike Lueken, Isabel Dziobek, Martha Nari Havenith, Marie Schild, Laura Stefanie Daedelow

**Affiliations:** 1 Department of Psychology, Humboldt-Universität zu Berlin, Berlin, Germany; 2 German Center for Mental Health (DZPG), partner site Berlin-Potsdam, Berlin, Germany; 3 Zero-Noise Lab, Ernst Strüngmann Institut für Neurowissenschaften, Frankfurt a. M., Germany

**Keywords:** Diseases, Medical research, Neuroscience, Psychology, Psychology

## Abstract

High ventilation breathwork (HVB) can induce altered states of consciousness with phenomenological similarities to those reported following administration of serotonergic psychedelics such as lysergic acid diethylamide (LSD), psilocybin or ayahuasca. Non-pharmacological methods capable of reliably eliciting such states may provide scalable models for investigating therapeutic mechanisms associated with psychedelic experiences while avoiding pharmacological and regulatory constraints. However, the therapeutic relevance and risk–benefit profile of HVB remain insufficiently characterized. In this exploratory study, 48 mental health professionals participated in an HVB workshop and evaluated perceived therapeutic potential, risks, and potential clinical indications. Participants reported altered states reaching intensity ranges comparable to those described in studies using moderate doses of serotonergic psychedelics. Internalizing disorders were suggested as indications, whereas psychotic, dissociative, and bipolar disorders were identified as contraindications. T-tests showed that perceived clinical potential of HVB significantly outweighed anticipated risks. Exploratory regression analyses indicated that trait openness and emotional breakthrough predicted perceived potential of HVB. HVB may represent a feasible non-pharmacological psychedelic induction paradigm warranting evaluation in controlled clinical trials.

## Introduction

Recent advances in psychedelic therapy have renewed interest in the therapeutic role of altered states of consciousness^1,2^. While serotonergic psychedelics such as lysergic acid diethylamide (LSD), psilocybin or ayahuasca reliably induce such states, their clinical implementation remains constrained by regulatory, financial, and safety considerations. An emerging question is therefore whether therapeutically relevant psychedelic-like experiences can be induced through non-pharmacological means. Behavioral induction methods for altered states of consciousness such as high ventilation breathwork^3^ (HVB) offer a unique opportunity to experimentally and clinically investigate core therapeutic processes - including emotional breakthrough^4^, mystical-type experiences^5^, and experiential insight^6^ - independently of drug administration. Establishing validated non-pharmacological induction paradigms may complement pharmacological approaches by enabling scalable research models and increasing accessibility of psychedelic-informed interventions.

Breathwork comprises a variety of practices that involve intentional manipulation of the breath (e.g., fast, deep cycles or slow-paced breathing) in order to promote relaxation and a sense of physical, mental and emotional well-being^7^. The potential of breathwork practices to relieve symptoms of psychological distress is suggested by a long cultural tradition with complex historical roots in Buddhism and psychedelic communities^3,8,9^. Slow-breathing techniques as treatments for mental disorders have been well researched^10^. In contrast, HVB practices such as Rebirthing Breathwork, Holotropic Breathwork®, Sudarshan Kriya Yoga (SKY), Wim Hof Method (WHM) and Conscious-Connected Breathing (CCB) shifted into focus of clinical research only recently^3,11–14^. Interest has been particularly sparked by repeated reports of intense psychological effects (e.g., emotional breakthrough, insight, catharsis^3,9^) that parallel the acute effects underlying pharmacologically-induced psychedelic therapies with serotonergic psychedelics^11,14,15^ - but with comparably lower legal and regulatory hurdles and a more beneficial safety profile.

Contemporary applications of HVB consist of prolonged periods of hyperventilation (several hours in Holotropic Breathwork®, 30–60 min in CCB). HVB is usually conducted in group settings while laying down in the presence of facilitators and accompanied by emotionally evocative music (see supplemental Fig. S1 for an illustration). Psychoeducative information and intention setting prior to the experience as well as integration efforts after the experience are of key importance both for participant safety and for maximizing the (postacute) effects of the intervention. HVB can be generally considered safe when controlling for physical exclusion criteria such as epilepsy, neurological, cardiovascular or respiratory diseases and pregnancy^3^. While severe mental disorders such as psychosis are also treated as exclusion criteria^3^, there is a lack of knowledge on the relative indications and contraindications with regard to other mental disorders. For example, initial positive results have been reported for certain HVB techniques such as SKY and yogic-type HVB in military veterans suffering from post-traumatic stress disorder (PTSD)^16–18^. However, caution is warranted, as hyperventilation can induce dissociation^3^ (which is a central pathological feature of PTSD) particularly in vulnerable subjects^19^. Hyperventilation can furthermore cause physical symptoms such as tingling, tetanic spasms, dizziness, or an increased heart rate, which typically occur during panic attacks and episodes of intense anxiety. On the other hand, interoceptive exposure is a first-line treatment for panic disorder (PD), aimed at providing new insights into the actual level of danger posed by physical symptoms. Therefore, HVB for anxiety disorders may well include components of interoceptive exposure. As such, clarification is needed regarding whether PTSD and PD should be regarded contraindications or targets for specific HVB protocols.

Physiologically, HVB can be regarded as a neurometabolic intervention. It alters the O_2_ - CO_2_ balance^14,20^, resulting in respiratory alkalosis, which triggers a cascade of autonomic and central-nervous system changes^21–23^. Physical symptoms of respiratory alkalosis (e.g., tingling in the hands and face, light-headedness, feelings of warmth or cold, changes in heart rate, muscle tightness or cramping) are common, possibly contributing to subjective phenomena such as disembodiment and depersonalization. Respiratory alkalosis decreases oxygen supply to the brain through the Bohr Effect, augmented by selective restrictions in blood flow to cortical areas including the insula - a hub of interoception and emotional evaluation of sensations^22,24^. On the level of the brainstem, an altered O_2_-CO_2_ balance activates the raphe nuclei, followed by increased levels of central serotonin^25^, which may convey psychedelic experiences in a similar way as serotonergic psychedelic substances do^26^, although the mechanisms of action underlying psychedelic-like effects of HVB are still incompletely understood.

Psychologically, HVB can induce altered states of consciousness (ASCs) at levels comparable to those by moderate doses LSD, psilocybin or ayahuasca^14,22,27^. Practitioners report intense sensory and emotional phenomena, including feelings of disembodiment and depersonalization, transcendence of space and time, ego dissolution, mystical-type experiences and insightfulness, often culminating in a sense of relief or transformation^9^. ASCs, moreover, predict subsequent improvements in mood and well-being^14^. In turn, the Big 5 personality trait “openness” as well as intense emotional reactions subsumed under the term “emotional breakthrough” act as moderators of positive responses towards psychedelics^4,28,29^. Although profound evidence is yet missing on the biological foundation of ASCs, first studies show that a reduction in end-tidal CO_2_ pressure due to deliberate hyperventilation is significantly correlated to ASC onset^14^. Furthermore, acute ASCs during HVB were associated with neural alterations as measured by EEG and cerebral blood flow^22,23^, at least some of which resemble those evoked by psychedelic states^23^.

Clinically, the potential of HVB as a stand-alone treatment or adjunct to psychotherapy remains largely unexplored. Most studies are conducted on healthy or subclinical populations, with variable effects on dimensional outcomes such as stress, anxiety, or life satisfaction^3,12,13,30,31^. The feasibility and tolerability of breathing techniques in healthy populations seems to be acceptable with low dropout rates and low rates of undesired effects^13^, but most studies lack systematic reporting^3^. In clinical populations such as anxiety disorders, HVB has been rarely investigated and the few available studies lack methodological scrutiny^3,10,12^.

The present study aimed to investigate HVB a candidate non-pharmacological method for inducing psychedelic-like altered states of consciousness and to build a translational bridge into its application as a behavioral psychedelic induction paradigm for clinical populations. To address this question, mental health professionals participated in a structured HVB workshop within the HEART study (High Ventilation Breathwork for Therapists) and subsequently evaluated experiential intensity, anticipated clinical utility, and safety considerations. In addition, we explored whether individual characteristics and acute experiential factors - particularly personality traits and emotional breakthrough - were associated with perceived therapeutic potential. We hypothesized that the personality trait “openness”^28,32^ and emotional breakthrough experiences^4,29^ during HVB would predict higher perceived potential and lower risks, respectively. By systematically assessing expert-informed evaluations following direct experiential exposure, this study aims to provide an initial empirical framework for situating HVB within contemporary psychedelic research and to inform future controlled investigations of non-pharmacological psychedelic induction paradigms.

## Methods

### In- and exclusion criteria, recruitment pathway

As we aimed to assess professionals with experience in the treatment of clinical populations, we included participants both with a medical degree (e.g., psychiatrists) and degree in psychology (e.g., psychological psychotherapists), who were either board-certified or currently in advanced postgraduate training (already independently treating patients). Psychotherapeutic approaches encompassed specializations in cognitive behavioral therapy, psychodynamic and systemic therapy. Clinicians with training solely in children and youth mental health were excluded. Somatic exclusion criteria comprised a self-reported history of or current respiratory diseases (any issues that impair the ability to control breathing such as active/chronic respiratory infections, nasal congestion, cough, cold, fever, etc. or a shortness of breath, abnormally slow or fast breathing), cardiovascular diseases (heart disease,  hypo- or hypertension) and neurological conditions (epilepsy, seizures, strokes or cerebral aneurysms), pregnancy, suspected pregnancy, trying to conceive, or breastfeeding, regular use of psychoactive or cardiovascular medications, any other physical/mental health conditions or current life events that impair the ability to participate in HVB. A self-reported history of fainting, syncope or of panic disorder, psychotic episodes or schizophrenia, bipolar disorder, panic attacks in the last 3 months, and problems during previous breathwork sessions (e.g., fainting) also led to exclusion. We recruited via mailing lists of all associated partner clinics (in- and out-patient) of the Humboldt Universität zu Berlin, Berlin-based psychotherapeutic training institutes of different approaches, and mail-outs to shared practices in Berlin and HVB networks of the study team. A website was set up to inform interested participants and to register for their preferred date (https://breathwork-psychotherapie.de/). Written informed consent was obtained prior to participation. Participants were not compensated financially for their study participation. The research protocol was approved by the local Ethics Committee of the Humboldt Universität zu Berlin (application number 2024-63) and adhered to the Declaration of Helsinki.

### Study design and procedure

**Fig. 1:**
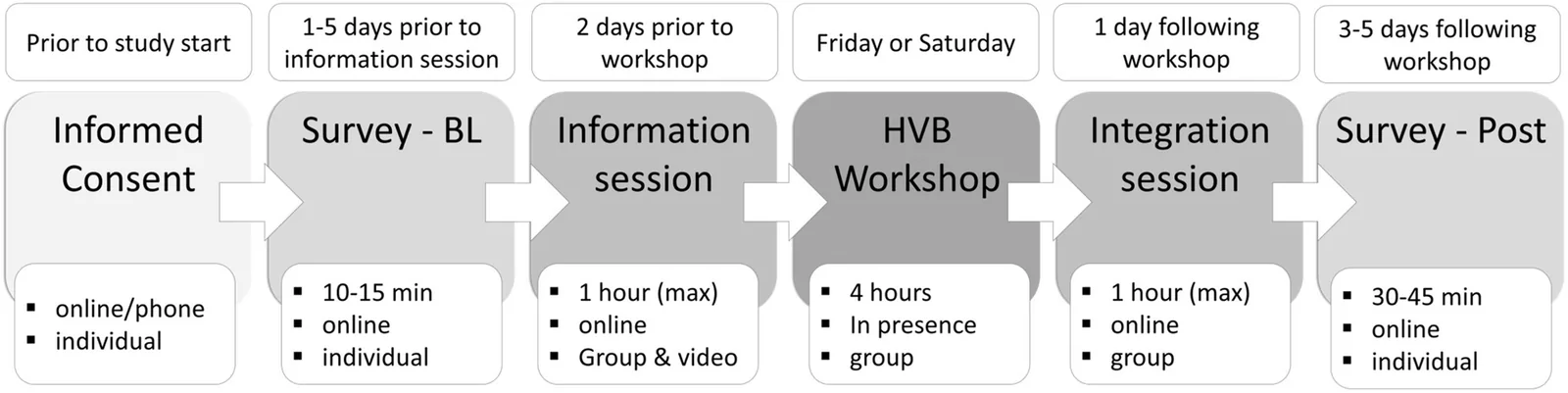
Study flowchart. BL baseline, HVB high ventilation breathwork.

We employed a one-group pre-post design to examine the subjective effects and clinical evaluation HVB in mental health professionals. Participants took part in a standardized HVB session as part of a structured workshop. No randomization or separate control condition was implemented. All participants received the same intervention. The study procedure is given in Fig. 1. During registration, participants were provided with the study information and the informed consent form via download. Additionally, they were presented with the list of exclusion criteria, asked to confirm that they read and understood these and that none applied to them. Upon arrival at the in-person HVB workshop, each participant was screened for the exclusion criteria again. The study procedure was set up to minimize information bias by providing standardized information on HVB (via a pre-workshop information video and online meeting), to adhere to the exclusion criteria (via online and on-site screening), and to support the post-acute integration of experiences to maximize the safety of all procedures (via a post-workshop online integration session). To provide pre-workshop information, participants were asked to watch a standardized information video and attend an online meeting, where they could get to know the facilitators of their workshop and the other participants and had the chance to ask questions regarding the information provided in the informed consent and the video. An online integration session was scheduled the morning after the workshop, led by the workshop facilitators, where participants were invited to share or discuss their experience in the group and bring up insights, concerns and questions in an open format. To ensure follow-up support if needed, the contact information of the facilitators was provided to every participant after completing the post assessment.

### HVB workshop: set and setting

The intervention protocol was specifically developed, manualized, and pilot-tested for a randomized controlled trial on participants with childhood trauma experiences, currently running at Humboldt-Universität (study protocol: osf.io/k3mab/overview). In contrast to breathwork formats commonly used in paramedical settings, the procedure was systematically adapted to meet research-grade safety and transparency standards, including a structured preparatory videoconference two days prior to the session, a next-day integration session, and explicit, domain-specific consent procedures (e.g., for physical touch). Between November 2024 and January 2025, we conducted six four-hour HVB workshops with a total of 54 participants. Workshops were carried out in an indoor martial arts school in the urban area of Berlin, Kreuzberg. The room ambience was curated by the study team using warm indirect lights and fabrics to increase comfortability, all mirrors were covered (photographs of the room are provided in the supplements S1; the authors have obtained written consent to publish images of the room where only HVB facilitators, but no participants are depicted). There was no direct access to nature. The room contained objects used in martial arts due to the usual classes taking place in the space. Eyeshades were provided to all participants to ensure sensory reduction if needed. Two separate bathrooms were accessible during the workshop. The workshop was designed to foster an empathic, respectful and safe relationship between participants and facilitators. None of the participants had a prior therapeutic relation with any of the facilitators. Set and setting are reported according to the ReSPCT guidelines for setting in psychedelic clinical trials^33^.

Each HVB workshop was conducted with a total of max. 15 participants attending. Four experienced facilitators were present, ensuring sufficient support for all participants. At the beginning and the end of the workshop, additional study personnel were present for logistical purposes and to welcome the participants. Facilitators were trained or certified in the breathing technique CCB and the applied therapeutic framework could be described as ‘non-directive’ or ‘supportive’. An individualized approach was utilized. For the workshop, participants and facilitators were positioned in a circle in the room facing each other. It was designed in consultation with an experienced HVB facilitator (MNH), who was also involved in the conceptualization and conduction of the study.

The workshop contained the following elements in consecutive order: introduction, mindfulness exercise, a sharing circle, the demonstration of the breathwork technique applied (including information on and consent to touch during active breathing), dynamic meditation and intention setting, an active (30 min) and a passive (20 min) phase of breathing, time for reflection and integration advise and a closing circle, resulting in a total of 4 hours including breaks (for detailed plan see supplemental Table S1). The breathing phase was accompanied by curated music playlists that had been used in other HVB workshops before. During the demonstration of the technique participants were informed about the use of physical support including touch by the facilitators during the session, and could indicate which level of support they would feel comfortable with during the breathing phase (support only when asked for explicitly, support only when evidently necessary, support based on intuitive assessment by the facilitator). At this point, participants were also explicitly informed of the possibility to ask for increased support if needed, and to decline facilitator support that felt unhelpful, and invited to practice open communication of their needs throughout the session.

No other medical or experimental procedures were performed during or in direct timely proximity of the session. Before the start of the workshop, a 30 min time slot was planned for arrival and time to decompress from all previous activities with a small lunch snack, fruits, cold and warm drinks being provided. Participants were instructed not to plan immediate subsequent activities after the session. To maximize safety and foster the post-acute integration of experiences, the workshop was always held on Friday or Saturday afternoons, not interfering with work-related demands.

### HVB workshop: breathing protocol

The breathwork session itself spanned 50 min, including an active breathing phase of approx. 30 minutes and a relaxed breathing phase of approx. 20 min. The format was largely based on a typical CCB session. Participants were asked to breathe through their mouth, connecting long, deep inhales (‘belly breaths’) with relaxed, passive exhales (‘sighs’). The aim was to eliminate pauses between in- and exhales, resulting in a pattern of continuous (‘connected’ or ‘circular’) breathing at a slightly faster rate than in everyday life. Participants practiced these breathing patterns before the session, but were also advised that if experiences during the breathwork session (e.g., the urge to laugh or cry) were to interrupt the recommended breathing pattern, this was welcome. Most participants independently slowed down their breath to a normal pace after approx. 30 min when the accompanying music became more restful. If participants were still breathing actively after 35–40 min, they were verbally invited by facilitators to slow down their breathing. Approximately 5 min before the end of the session, participants were invited to bring their experience to a close in their own time.

### Assessments

Assessments were conducted online using SoSciSurvey 3.8.00. At baseline, we collected participant information on sociodemographics, clinical experience and previous experience on HVB as well as personality traits using the Big Five Inventory (BFI-10^34^). At post, we assessed the acute and subacute somatic and cognitive side-effects of HVB and how much participants rated their overall experience as unpleasant or pleasant indicating the intensity in percentage ranging from not at all (0%) to very strong (100%). Altered states of consciousness during HVB were assessed using a subset of 42 items of the Altered States of Consciousness Rating Scale^35^ (11-ASC) encompassing 11 factors of the acute altered states experiences (experience of unity, spiritual experience, blissful state, insightfulness, disembodiment, impaired control and cognition, anxiety, complex imagery, elementary imagery, audio-visual synesthesia, and changed meaning of percepts). All 11 scales can be assigned to one of the dimensions of an altered state of consciousness as defined by Studerus et al.^35^ as ‘Oceanic Boundlessness’, ‘Visionary Restructuralization’ and ‘Dread of Ego-Dissolution’. In particular, Oceanic Boundlessness captures core elements of mystical-type experience, such as unity with the self and others, blissful state and positively experienced depersonalization. Emotional breakthrough experiences were assessed as a potential therapeutic mechanism of change in psychedelic therapy using the Emotional Breakthrough Inventory^4^ (EBI). This 6-item inventory assesses emotional breakthrough during a psychedelic experience (“I faced emotionally difficult feelings I usually push aside” as an exemplary item). Items of the 11-ASC and EBI were rated using a visual analogue scale (VAS 0–100) with higher values indicating more intense experiences. The clinical evaluation of HVB encompassed the assessment of its therapeutic risks and potential (“How do you assess the risks/the potential of breathwork in a therapeutic context?”), each respectively ranked on a VAS from 0 to 100. These were followed by open text formats to further elaborate on the perceived risks and potential of this method which were analyzed qualitatively. A list of most common DSM-5-TR diagnoses were shown and participants were asked to indicate whether the application of HVB for the treatment of the following diagnoses were promising or unsuitable, deriving a list of recommended indications and contraindications. Means per each diagnosis were calculated, ranging between –1 to +1.

### Statistical analysis

Sample characteristics (demographics, clinical qualifications and experiences, previous HVB experience, personality traits) and acute experiences under HVB (subjective somatic and cognitive effects, 11-ASC, EBI, overall (un-) pleasantness, perceived risks and potential) were calculated as number (percentage) or means (standard deviations). Differences between (un-) pleasantness, risks and potential were tested using dependent t-tests. To explore whether personality traits and the depth of emotional breakthrough experience predicted experts’ assessment of adjunct risks and potential of HVB, two exploratory linear regression models were conducted controlling for age and gender as potential confounders^35,36^ and personality traits as assessed by BFI-10 and emotional breakthrough experiences as assessed by the EBI. Analyses were carried out with the statistical package for the social sciences (SPSS 29.0) using a significance threshold of *p* < 0.05. Open-text responses on perceived risks and potential were thematically analyzed using MAXQDA Plus 24 (version 24.10.0). An iterative procedure that combined both deductive and inductive analysis approaches was carried out closely following the methodology of structuring qualitative content analysis described in ref. ^37^.

## Results

**Fig. 2:**
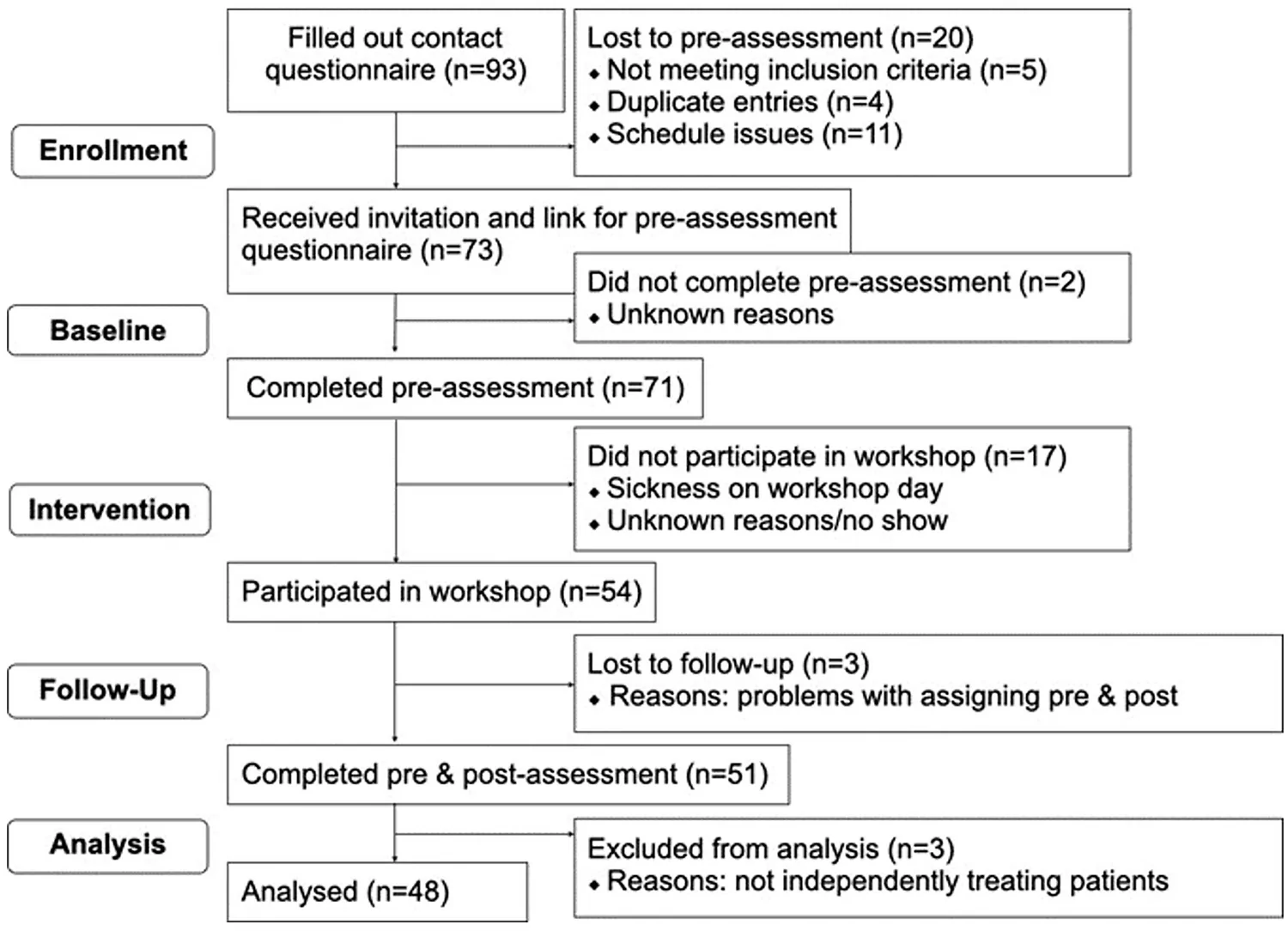
Participant flowchart.

A participant flowchart is presented in Fig. 2. Characteristics of the sample can be found in Table 1. The majority of participants were board-certified psychotherapists with clinical experience both with in- and outpatients of several years. More than half of the participants were breathwork-naive. Information on race and ethnicity of participants was not collected.

**Table 1:** Sample characteristics of participants, acute experiences and perceived therapeutic risks and potential of HVB (*n* = 48)

	*n* (%) or mean (SD)
Gender identification	
Female	36 (75.0)
Male	12 (25.0)
Other/n. a.	0 (00.0)
Age (years)	37.9 (7.6)
Clinical qualification	
License acquired	29 (60.4)
In Training	19 (39.6)
Clinical experience (years)	
Inpatients^1^	3.6 (4.3)
Outpatients	5.4 (4.2)
Type of completed professional qualification	
Medical degree with subsequent specialization in Psychiatry and Psychotherapy	2 (4.2)
Medical degree with subsequent specialization in Psychosomatic Medicine and Psychotherapy	1 (2.1)
Medical degree with additional (specialized) training in Psychotherapy	1 (2.1)
Training in Psychological Psychotherapy with a focus on Cognitive Behavioral Therapy	32 (66.7)
Training in Psychological Psychotherapy with a focus on Psychodynamic Psychotherapy	8 (16.7)
Training in Psychological Psychotherapy with a focus on Systemic Therapy	3 (6.3)
Other type of training	2 (4.2)
Diagnosis-specific clinical experience (yes)	
Generalized anxiety disorder	7 (14.6)
Panic disorder	14 (29.2)
Social anxiety disorder	12 (25)
Specific phobias	2 (4.2)
Obsessive-compulsive disorder	6 (12.5)
Body dysmorphic disorder	1 (2.1)
Post-traumatic stress disorder	14 (29.2)
Dissociative disorders	-
Somatic stress disorders and related disorders	2 (4.2)
Sleep-wake disorders	-
Eating disorder	2 (4.2)
Bipolar disorder	2 (4.2)
Depression	41 (85.4)
Schizophrenia or other primary psychoses	6 (12.5)
Substance-related dependency	6 (12.5)
Behavioral addictions	2 (4.2)
Impulse control disorders	2 (4.2)
Personality disorder	12 (25)
Autism spectrum disorders	2 (4.2)
Attention-deficit/hyperactivity disorder (ADHD)	1 (2.1)
Couple and family problems	2 (4.2)
Prior breathwork experience (yes)	20 (41.7)
Personality: Big Five Inventory	
Extraversion	3.6 (0.8)
Agreeableness	3.5 (0.6)
Conscientiousness	3.7 (0.8)
Neuroticism	3.0 (0.9)
Openness	4.0 (0.7)
Emotional Breakthrough Inventory (0–100 VAS)	51.9 (23.8)
Overall unpleasantness HVB (0–100 VAS)	23.6 (20.1)
Overall pleasantness of HVB (0-100 VAS)	60.7 (25.8)
Perceived psychotherapeutic risks (0–100 VAS)	28.0 (18.1)
Perceived psychotherapeutic potential (0–100 VAS)	68.4 (19.2)

^1^ available for *n* = 46 participants, HVB: high ventilation breathwork.

### Acute and subacute effects of HVB

**Fig. 3:**
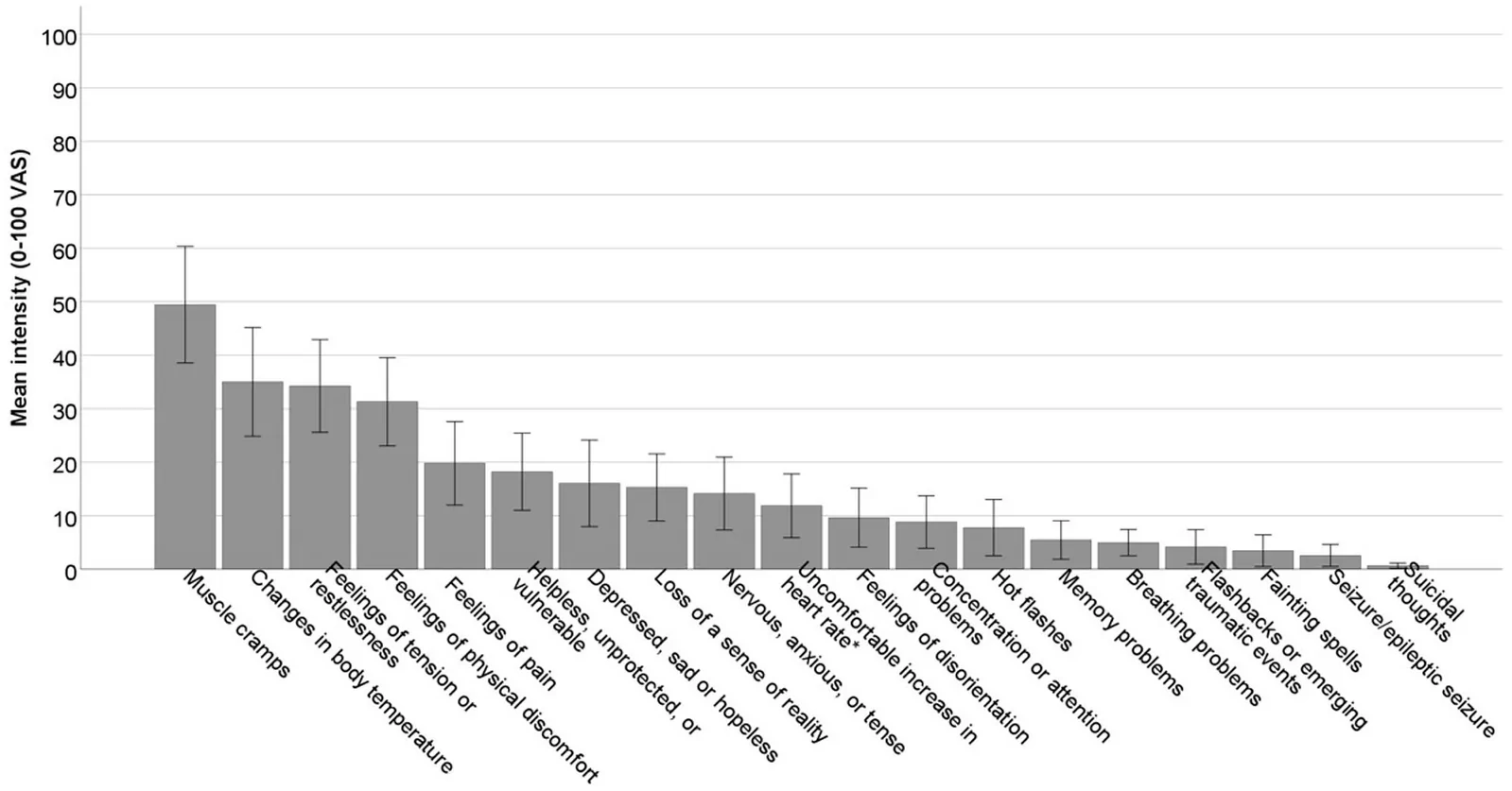
Acute and subacute somatic and cognitive effects of high ventilation breathwork. Given are mean intensity values for *n* = 48 participants. For effects indicated with *n = 47. Error bars indicate the standard error of means (SEM). VAS visual analogue scale.

Acute and subacute somatic effects with the highest reported intensity were muscle cramps, changes in body temperature and feelings of tension or restlessness. Cognitive effects were reported with less intensity than somatic effects with loss of sense of reality, feelings of disorientation and concentration or attention problems being the most commonly reported. Suicidal thoughts were reported the least. Mean reported intensity per effect is presented in Fig. 3. The overall pleasantness of the HVB experience significantly outweighed unpleasantness ratings (*t* (47) = 5.87, *p* < 0.001; see Table 1 for details).

**Fig. 4:**
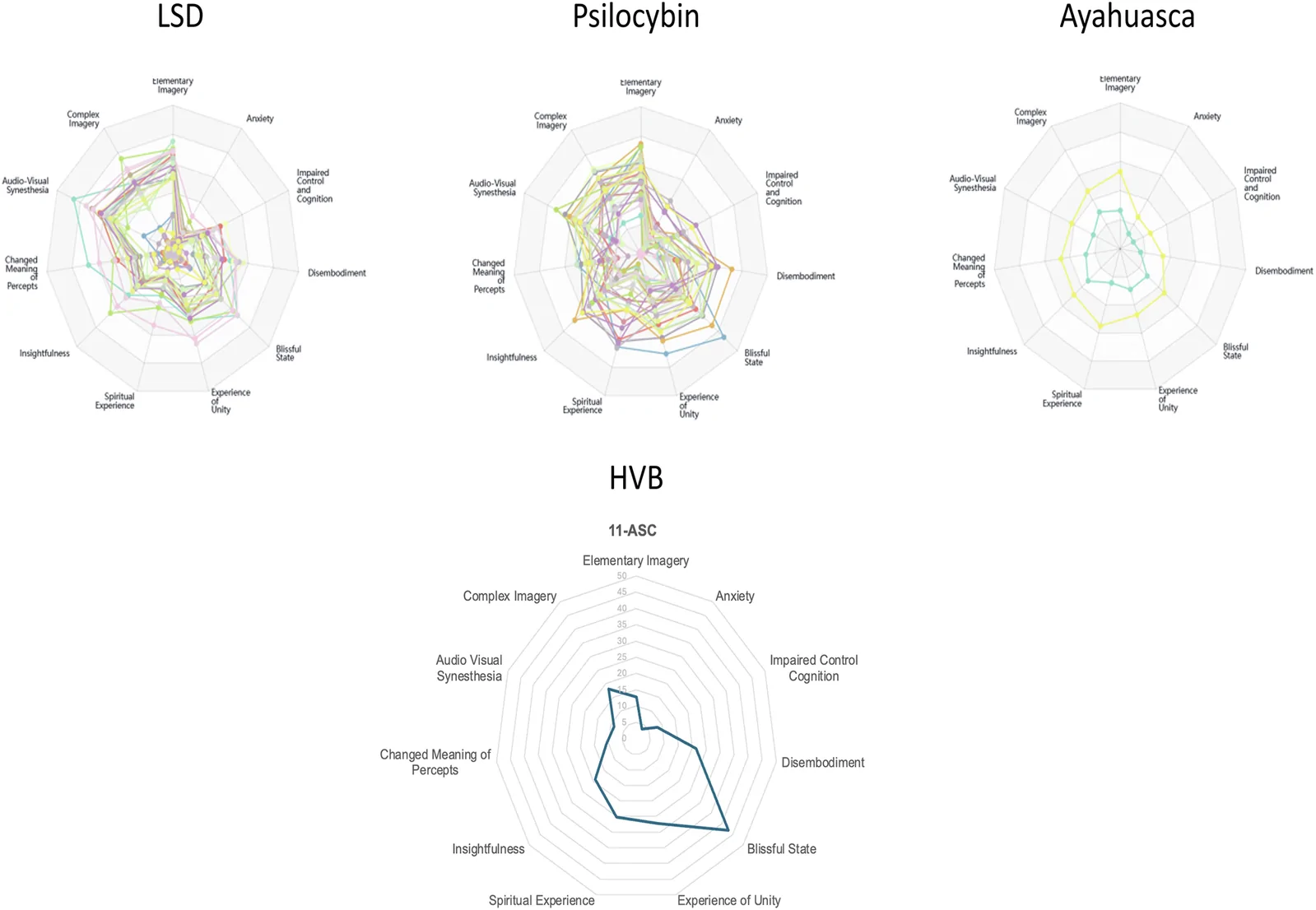
Radar chart of altered states of consciousness of high ventilation breathwork. Given are the means of the 11 factors of the Altered States of Consciousness Scale (revised) as assessed by the 11-ASC. Upper line: Descriptive comparison with studies on Lysergic Acid Dietylamide (LSD; *n* = 38 studies); psilocybin (*n* = 31 studies) and ayahuasca (*n* = 2 studies; retrieved from https://alteredstatesdb.org/). Lower line: results after high ventilation breathwork (HVB; *n* = 48 participants).

Altered states of consciousness as assessed by the 11-ASC showed the highest loads on blissful state, experience of unity and spiritual experience. Factors with the lowest loads were anxiety, impaired cognitive control and audio-visual synesthesia. Figure 4 shows the radar chart of the means of the ASCs during HVB. Since our study did not have a control condition, we plotted the subjective experience during HVB alongside the dose-dependent relationships of LSD, psilocybin, and ayahuasca-induced subjective experiences^38^. We did not conduct a quantitative comparison given that these data stem from independent studies.

### Clinical evaluation of HVB

**Fig. 5:**
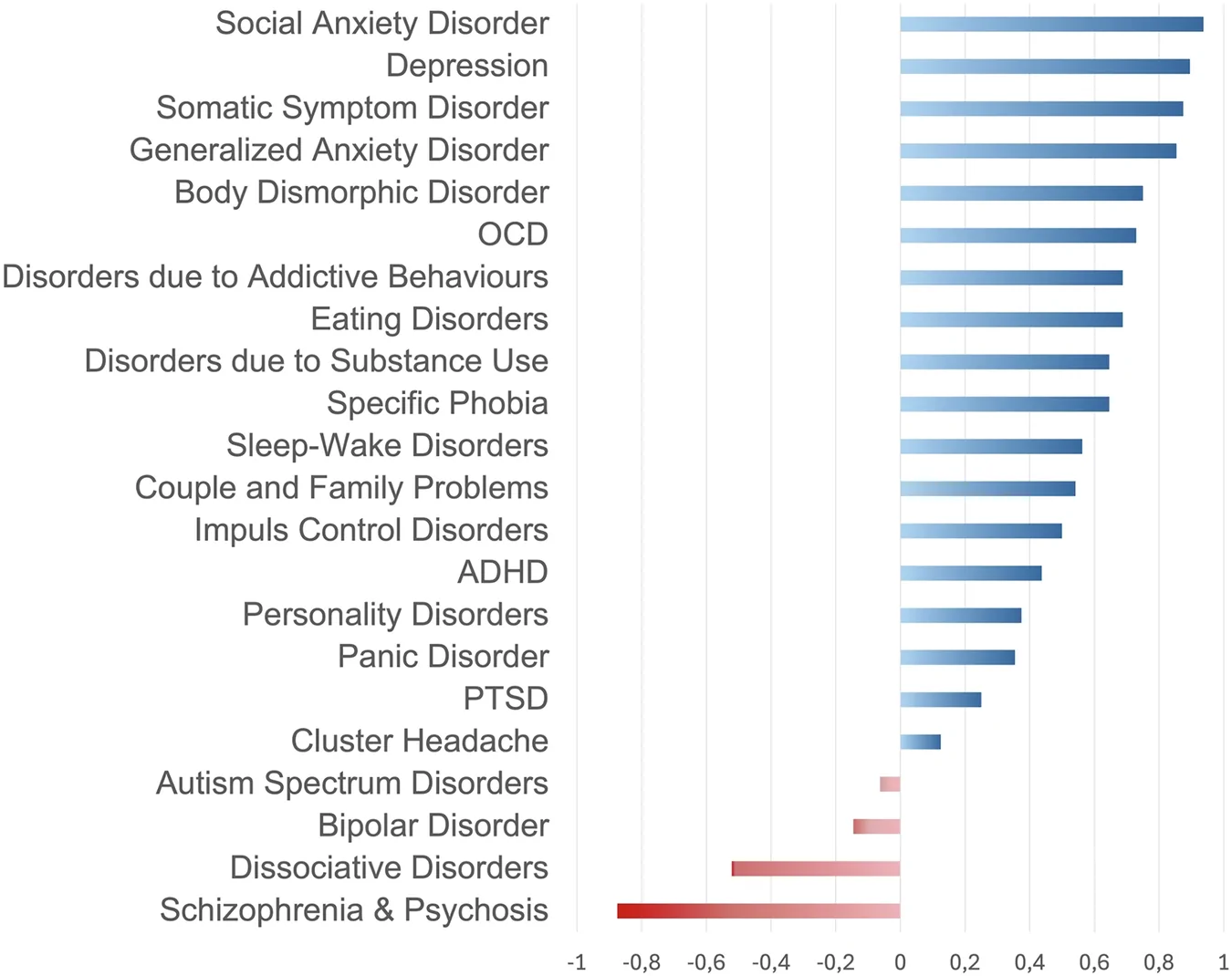
Clinical recommendation on (contra-) indications for high ventilation breathwork. Given are means of indications (blue) and contraindications (red) for mental disorders according to the Diagnostic and Statistical Manual of Mental Disorders fifth version text revision (DSM-5-TR). OCD obsessive compulsive disorder, ADHD attention deficit hyperactivity disorder, PTSD post-traumatic stress disorder.

Participants’ recommendations on indications and contraindications of HVB are shown in Fig. 5. The clinical recommendation of HVB was highest for social anxiety disorder, depression, somatic symptom disorder and generalized anxiety disorder, but also recommended a range of other (primarily internalizing) conditions. Schizophrenia & psychosis, dissociative, bipolar and autism spectrum disorder were perceived as contraindications. Recommendations on PTSD and panic disorder were lower as compared to other internalizing disorders.

The perceived therapeutic potential of HVB significantly outweighed the risks (*t* (47) = 9.36, *p* < 0.001; see Table 1). Two multiple linear regression analyses including age, gender, the five personality traits extraversion, agreeableness, conscientiousness, neuroticism and openness as well as the EBI score indicating the depth of emotional breakthrough experiences were conducted in order to predict perceived risks and potential (for full results on the regression analyses please see supplemental Tables S2 and S3). For potential, the model tested significantly (*F* (8, 39) = 2.57, *p* = 0.024), explaining approximately 35% of the variance (R^2^ = 0.35, adj. R^2^ = 0.21). Older age (beta = 0.89, *SD* = 0.35, *t* = 2.53, *p* = 0.016), higher openness (beta = 9.82, *SD* = 3.75, *t* = 2.62, *p* = 0.013) and steeper emotional breakthrough experiences (beta = 0.23, *SD* = 0.11, *t* = 2.12, *p* = 0.041) significantly predicted higher perceived potential, while the remaining personality traits showed no significant effects. Regarding perceived risks, the overall model showed a non-significant trend (*F* (8, 39) = 2.07, *p* = 0.063), explaining approximately 30% of the variance (R^2^ = 0.30, adj. R^2^ = 0.15).

Thematic analyses of open text formats on perceived risks and potential were derived from responses from *n* = 45 (95.8%) participants reflecting on perceived potential and *n* = 43 (89.6%) discussing risks. For potential, six key themes were identified: emotional processing and regulation (*n* = 31, 68.9%), cognitive and consciousness expansion, body-oriented or somatic effects (*n* = 20, 44.4%, each), self- and resource strengthening (*n* = 17, 37.8%), exposure (*n* = 8, 17.8%), and trauma-oriented effects (*n* = 5, 11.1%). Regarding the risks of HVB, responses clustered thematically around six key themes: psychological destabilization and symptom exacerbation (*n* = 27, 62.8%), risks due to therapeutic setting and patient boundaries (*n* = 10, 23.3%), lack of support or insufficient follow-up care (*n* = 8, 18.6%), risk of dissociation (*n* = 6, 14.0%), addictive potential of HVB and spiritual bypassing (*n* = 3, 7.0%, each) and a category in which risks were not seen as substantial (*n* = 3, 7.0%). Text examples in each category are given in the supplemental Table S4.

## Discussion

Interest in HVB as a behavioral tool for inducing psychedelic experiences and improving mental health and well-being has grown significantly in recent years^3^. Since rigorous clinical research on this technique is still in its infancy, we wanted to explore the perspectives of mental health professionals on HVB as a potential therapeutic tool. Our study yielded the following key findings: (a) acute effects were predominantly benign, with mild side effects and moderate psychedelic experiences, mainly loading on the factor “oceanic boundlessness”; (b) clinicians provided clear recommendations on indications and contraindications, with a particular focus on use for internalizing disorders and additional caution to be warranted for severe mental disorders, including psychosis and bipolar disorder; (c) clinicians’ age and the personality trait of openness, as well as emotional breakthrough experiences positively predicted  evaluations of perceived potential; and (d) the description of risks and potential pointed to transdiagnostic, process-based mechanisms of action, but also to challenges for both therapists and patients that need to be considered in future clinical applications of this technique.

HVB as a neurometabolic intervention is associated with the known somatic effects of sustained hyperventilation, such as muscle cramps, changes in body temperature, and feelings of physical tension, restlessness, or discomfort^3^. This was also the case in our sample, although the symptoms were rated as moderately intense and, overall, the pleasantness of the experience outweighed the unpleasant feelings. However, it should be noted that about half of the participants were not breathwork-naive; therefore, caution is advised when using HVB with clinical populations who have no prior experience and may be particularly susceptible to negative experiences with somatic and cognitive side effects (e.g., anxiety disorders). Careful patient selection and comprehensive advanced information about the expected somatic and subjective effects are essential for vulnerable groups and should be included in the standard repertoire for preparing patients who wish to undergo HVB, as well as expanding preparation and integration of experiences in clinical groups where mere online sessions might not be sufficient.

We propose HVB as a behavioral induction paradigm into psychedelic therapy. The psychedelic effects of HVB appear to be a reliable phenomenon that has been reported for different target groups and is comparable to moderate doses of serotonergic psychedelics^11,14,22,27^. Supporting previous findings, participants in our study reported moderate psychedelic effects on established questionnaires, mainly scoring high on the dimension of “oceanic boundlessness,” which is associated with mystical-type experiences that are discussed as a key mechanism of psychedelic therapy^5,36^. This pattern was very similar to comparison data from studies using LSD, psilocybin and ayahuasca (albeit only few studies were available on the latter), with similar responses on the factor “oceanic boundlessness”, while manifestations of complex imagery, elementary imagery, audio-visual synesthesia were lower for HVB as compared to serotonergic psychedelics. In contrast to serotonergic psychedelics, the psychedelic effects of HVB can be easily adapted or terminated by interrupting voluntary hyperventilation. These properties (moderate intensity and possibility of titration/termination) make HVB particularly suitable for patients with no previous psychedelic experience, familiarizing them with psychedelic therapy as a first step. In addition, trying out HVB may facilitate informed consent to substance-based psychedelic therapy, where the ineffability of psychedelic experiences poses a significant ethical problem^39^.

Mental health professionals provided clear recommendations on contraindications related to mental illnesses, including psychosis and schizophrenia, dissociative disorders, bipolar disorders, and (to a lesser extent) autism spectrum disorders. These recommendations overlap in part with published recommendations based on anecdotal evidence (e.g., psychosis and dissociative states; see refs. ^3,9^), but also add bipolar disorder to the list of suspected contraindications, possibly due to the perceived increased risk of triggering (hypo-) manic episodes. Pharmacologically induced psychedelic therapy is currently also tested for a wide range of mental disorders, including (treatment-resistant) depression, substance-use disorders, anxiety disorders, obsessive-compulsive disorders and PTSD^40^, showing some overlap with professionals’ recommendations on HVB. Although HVB techniques such SKY and yogic-type HVB have been used successfully in military veterans suffering from post-traumatic stress disorder (PTSD)^16–18^, clinicians’ recommendations in our study for the use of HVB in PTSD were rather low, and clear contraindications were identified for dissociative disorders, which may overlap with PTSD. However, it should be noted that SKY uses cyclic rhythmic breathing with different cycles ranging from slow-medium to fast-very fast and is therefore not directly comparable to the CCB format we used. Another issue concerns the use of HVB in PD. The “false alarm theory of suffocation”^41^ suggests that the detection of chemosensory changes in PD is hypersensitive and can be triggered by CO_2_, as demonstrated by the panic-inducing effects of breathing exercises such as CO_2_ inhalation^42,43^ (which leads to hypercapnia). Hyperventilation (leading to hypocapnia) can be seen as a compensatory mechanism for a hypersensitive CO_2_ detection system^3^. (Short phases of) hyperventilation as part of interoceptive exposure is already a regular component of PD treatment. Our findings support previous assumptions that caution should be exercised when using HVB in PD patients^3^, although the potential of using HVB as an interoceptive exposure tool may be the subject of future studies if patient safety is closely monitored. In summary, the present findings confirm known contraindications but also highlight new disorders that should be treated with caution in the context of HVB (e.g., bipolar disorder, autism spectrum disorder).

According to the current state of the literature, expert recommendations regarding PTSD and PD are not clear. Caution is advised when using HVB techniques in these clinical populations until more evidence on HVB’s safety and efficacy for PTSD and PD is available. At the same time, clinicians cited a wide variety of possible indications (including social anxiety disorder, generalized anxiety disorder, depression, or obsessive-compulsive disorder) that could benefit from HVB, thus opening up new targets for future clinical studies. As the physical symptoms of anxiety (and hyperventilation) are not as pronounced in other anxiety disorders, such as social anxiety or generalized anxiety disorder, as compared to PD, these indications may exert a more beneficial risk profile for HVB, which was reflected in the clinicians’ evaluations of (contra-) indications. These initial recommendations, based solely on clinical expertise, must now be supplemented by empirical research on the clinical effects of HVB.

Overall, the perceived potential outweighed the risks of HVB as a therapeutic tool. In line with our hypotheses, benefits were significantly predicted by the personality trait “openness” and steeper breakthrough experiences during the HVB session. In addition, age appeared as a third predictor in the regression analysis in that older age predicted higher perceived potential. Contrasting our initial hypothesis, we did not observe a significant prediction of the perceived risks. Present findings partly corroborate the relevance of personality traits and in-session processes such as emotional breakthrough experiences not only as important moderators of the effects of psychedelics^4,28,29,32^, but point toward therapists’ characteristics moderating the perceived clinical potentials of HVB. They could guide the selection of therapists who may consider this method as particularly useful in their therapeutic practice.

Findings on open text formats on perceived risks and potential indicated six different subject areas that can be integrated into contemporary psychotherapeutic approaches. With regard to potential, a large majority described intense *emotional processing and regulation* as a key positive effect. HVB was described as particularly valuable for individuals who tend to avoid and intellectualize emotions that often remain inaccessible in traditional (talk-based) therapeutic approaches. From a therapeutic perspective, HVB may recruit mechanisms similar to those of emotion-focused therapy^44^ (EFT) and resembles traditional concepts of catharsis. *Cognitive or consciousness expansion* was cited as a second potential, which can promote cognitive flexibility, interrupt maladaptive cognitive loops, and reduce cognitive rigidity^30^, which is a characteristic feature of many mental disorders and is typically addressed by cognitive therapy. The psychological insight resulting from this process is also discussed as a key mechanism in psychedelic therapy^6^. Furthermore, participants noted that HVB may facilitate access to material that normally underlies psychological defense mechanisms and thus remains outside of consciousness and, consequently, outside of verbal expression. As such, it can promote access to unconscious material as a key target in psychoanalytic therapy. *Body-oriented effects* of HVB were related to interoceptive exposure, in which confrontation with distorted bodily sensations can promote new learning and mastery. *Self- and resource strengthening* was considered helpful for restoring inner stability and a sense of safety, as it enhances patients’ sense of self-efficacy, self-confidence, and self-compassion. *Exposure* (counteracting avoidance of experiences, emotions, and bodily sensations) during HVB was highlighted as an additional potential, mirroring the exposure techniques and inhibitory learning approaches in cognitive-behavioral therapy and supporting recent findings from our group that reduced experiential avoidance during HVB supports psychological wellbeing after the session^45^. Finally, *trauma-oriented effects* were attributed to the somatic pathways that HVB may open up for reactivating and integrating fragmented traumatic memories. Embodiment approaches also emphasize the importance of somatic awareness, body memory, and mind-body connections, especially in relation to trauma^46,47^. In summary, HVB may activate different mechanisms of action that can be interpreted within the framework of modern process-oriented psychotherapy^48^. As a transdiagnostic treatment tool, it could transcend traditional diagnostic boundaries and bridge different therapeutic approaches.

Although the perceived potential of HVB outweighed the risks, more than half of the participants explicitly mentioned certain risks in the open text formats. These were (in descending order) psychological destabilization or worsening of symptoms, problems with boundary violations (increased suggestibility, lack of neutrality and therapeutic distance, and the danger of abuse of power by therapists), possible lack of support or inadequate aftercare, risk of malignant dissociation, as well as the addictive potential of HVB and the risk of spiritual bypassing (e.g., using spiritual practices as avoidance or defense mechanisms to circumvent unresolved issues). These results underscore the relevance of specific patient safety measures in relation to HVB. These include (i) careful selection of patients, taking into account somatic and psychiatric exclusion criteria, (ii) adequate preparation and consent prior to HVB, (iii) awareness of the ethical implications of increased suggestibility and therapeutic touch, and (iv) integration of HVB into psychotherapeutic (follow-up) care. When these specific patient safety considerations are taken into account, HVB offers potential as a novel adjunct to psychotherapeutic treatments.

Although the HEART study has several strengths (high degree of safety, standardization and documentation of the HVB workshop, use of state-of-the-art questionnaires from psychedelic research), comparisons with psychedelic studies are indirect and based on cross-study phenomenological benchmarks rather than controlled study designs. The present findings result from the entire workshop protocol, including preparation, group experience of HVB with evocative music, sharing, and integration and cannot be solely tracked down to the isolated effects of the breathing phase itself. It is well known that “set and setting” factors profoundly influence psychedelic experiences. Therefore, the reported subjective effects cannot be interpreted as attributable solely to the breathing technique itself. A mechanistic investigation of the isolated effects of HVB was beyond the scope of the present study, since our goal was to evaluate the potential and risks of the entire intervention protocol as it would be implemented in clinical practice. However, new findings suggest that altered CO_2_ levels are associated with psychedelic experiences^14^, making it likely that some of the variance can be attributed to the breathing component. The choice of mental health professionals is selective, but ecologically relevant to psychedelic therapy itself. Their origin from the urban area of Berlin, where greater openness to HVB is to be expected, may imply a trend toward optimistic evaluations. As a substantial proportion of therapists were not breathwork-naïve, caution is warranted regarding a too optimistic evaluation of the method. Previous experiences with psychedelics were not recorded and may have influenced both acute experiences and therapeutic evaluation. Future studies could additionally include professionals from other locations in Germany and internationally, and screen for experiences both with HVB and psychedelic substances. Subjective effects were assessed subacutely on the following days, which may have led to memory bias. No additional measures were included to investigate the mechanisms through which HVB can exert its effect. It should also be noted that the regression analyses are limited by the relatively small sample size, which could undermine confidence in the stability of individual regression coefficients. Furthermore, both outcome and process variables were combined, so the conceptual relationship is likely more complex than it would be with independent predictors. The results should be considered preliminary until they have been replicated in independent samples. Furthermore, future studies should include longer follow-up periods to investigate the subacute positive (e.g., afterglow phenomena, which are well described for psychedelic substances, see ref. ^49^) and negative effects of this technique.

In conclusion, HVB may represent a scalable behavioral model for investigating therapeutic processes associated with psychedelic experiences under conditions of reduced regulatory burden. HVB is a candidate non-pharmacological induction paradigm for psychedelic therapy that could clinically benefit a wide range of mental disorders from the internalizing spectrum. As such, our study contributes to ongoing efforts to improve accessibility, experimental tractability, and global scalability of psychedelic-informed mental health interventions. To ensure patient safety, certain measures must be taken with regard to adequate patient selection and preparation, ethical conduct, and psychotherapeutic integration. Appropriate therapeutic application could open up new avenues for HVB, both as a tool to better understand the mechanisms of action of psychedelic therapy and as a promising new treatment method for suffering patients.

## Supplementary information

Supplementary Information

## Data Availability

All data supporting the findings of this study are available within the paper and its Supplementary Information or are available upon request.

## Code availability

The data analysis code is available upon request.
