# Supplementary material for: High ventilation breathwork as a non-pharmacological model for inducing psychedelic-like states: expert evaluations of therapeutic potential and risk
Source: Npj Ment Health Res. 2026 Sep 17;5:48. doi: 10.1038/s44184-026-00246-x (PMC13586335; doi:10.1038/s44184-026-00246-x)
Supplement: Supplementary file 1 [file 44184_2026_246_MOESM1_ESM.docx]

**High ventilation breathwork as a non-pharmacological model for inducing psychedelic-like states: expert evaluations of therapeutic potential and risk**

Ulrike Lueken^1,2*^, Isabel Dziobek^1,2^, Martha Nari Havenith^3^, Marie Schild^1^, Laura Stefanie Daedelow^1,2^

^1^Department of Psychology, Humboldt-Universität zu Berlin, Germany

^2^German Center for Mental Health (DZPG), partner site Berlin-Potsdam, Germany

^3^Zero-Noise Lab, Ernst Strüngmann Institut für Neurowissenschaften, Frankfurt a.M., Germany

# **Supplemental Information**

**Supplemental Figure S1: Photographs of the workshop setting.**

|  |  |  |
| --- | --- | --- |

Supplemental Figure S1 depicts the setting characteristics of the venue where the high ventilation workshop was given. *Annotation:* People depicted in the photographs are facilitators and not participants. Permission for publication was provided.

**Supplemental Table S1.** Detailed plan of the guided breathwork session^1^.

| **Element** | **Explanation** | **Duration** |
| --- | --- | --- |
| Introduction | Explanation of the breathing method and the process | 15 minutes |
| Shaking & Mindfulness Exercise | Physical relaxation and concentration on the breath | 15 minutes |
| Group Sharing | Sharing initial impressions and expectations | 30 minutes |
| Break |  | 15 minutes |
| Demonstration of Breathwork | Demonstration of the breathing technique including consent to touch | 20 minutes |
| Dynamic Meditation & Intention Setting | Movement and mental preparation for the breathwork experience | 30 minutes |
| Breathwork | 30 minutes of active breathing, followed by 20 minutes of quiet reflection | 50 minutes |
| Silent Break |  | 15 minutes |
| Optional Group Sharing | Voluntary reflection on the experience | 25 minutes |
| Integration Advice | Tips for integrating the experience into daily life | 10 minutes |
| Closing Circle |  | 5 minutes |
| ^1^The intervention protocol was specifically developed, manualized, and pilot-tested for a randomized controlled trial on participants with childhood trauma experiences, currently running at Humboldt-Universität (study protocol: osf.io/k3mab/overview) | | |

|  | **Supplemental Table S2.** Results of the linear regression model predicting perceived risks of HVB. | | | | | | |
| --- | --- | --- | --- | --- | --- | --- | --- |
| **Predictor** | | **b** | **SE(b)** | **β** | **t** | **p** |  |
| Intercept | | 80.88 | 32.50 | - | 2,49 | 0.017 |  |
| Gender (female) | | -14.43 | 6.44 | -0.35 | -2.24 | 0.031* |  |
| Age | | -0.06 | 0.35 | -0.01 | -0.02 | 0.986 |  |
| Extraversion | | 4.20 | 3.43 | 0.19 | 1.22 | 0.299 |  |
| Agreeableness | | 0.35 | 4.49 | 0.01 | 0.08 | 0.938 |  |
| Conscientiousness | | -1.72 | 3.15 | -0.8 | -0.54 | 0.589 |  |
| Neuroticism | | 0.17 | 3.05 | 0.01 | 0.56 | 0.956 |  |
| Openness | | -10.1 | 3.67 | -0.38 | -2.77 | 0.009** |  |
| Emotional breakthrough | | -0.09 | 0.10 | -0.11 | -0.82 | 0.417 |  |
| HVB: high ventilation breathwork. Personality factors according to the Big Five Inventory (BFI-10); Emotional breakthrough as measured with the motional Breakthrough Inventory (EBI). B = unstandardized regression coeﬃcient; SE = standard error; β = standardized regression coefficient. **p* < .05, ***p* < .01. | | | | | | |  |

| **Supplemental Table S3.** Results of the linear regression model predicting perceived potential of HVB. | | | | | | | |
| --- | --- | --- | --- | --- | --- | --- | --- |
| **Predictor** | | **b** | **SE(b)** | **β** | **t** | **p** |  |
| Intercept | | -45.18 | 33.32 | - | -1.36 | 0.183 |  |
| Gender (female) | | -0.58 | 6.60 | -0.01 | -0.09 | 0.931 |  |
| Age | | 0.89 | 0.35 | 0.35 | 2.53 | 0.016* |  |
| Extraversion | | 2.56 | 3.52 | 0.11 | 0.73 | 0.472 |  |
| Agreeableness | | 4.27 | 4.60 | 0.12 | 0.93 | 0.359 |  |
| Conscientiousness | | 0.35 | 3.23 | -0.02 | -0.11 | 0.914 |  |
| Neuroticism | | 2.18 | 3.13 | 0.10 | 0.70 | 0.491 |  |
| Openness | | 9.82 | 3.75 | 0.35 | 2.62 | 0.013* |  |
| Emotional breakthrough | | 0.22 | 0.11 | 0.28 | 2.12 | 0.041* |  |
| HVB: high ventilation breathwork. Personality factors according to the Big Five Inventory (BFI-10); Emotional breakthrough as measured with the motional Breakthrough Inventory (EBI). B = unstandardized regression coeﬃcient; SE = standard error; β = standardized regression coefficient. **p* < .05. | | | | | | |  |
|  |  |  |  |  |  |  |  |

| **Supplemental Table S4.** Open text examples on perceived risks and potential of HVB by mental health professionals. | |
| --- | --- |
| Potential |  |
| Emotional processing and regulation | Emotional work can potentially be greatly intensified; access to emotions and further therapeutic work with/on them can be facilitated, especially in internalizing disorders. |
| Cognitive and consciousness expansion | I think breathwork can help break out of cognitive loops, dissolve feelings of emptiness or overwhelm, and potentially foster a curious view of one’s own body and mind through bodywork that can support the psychotherapeutic process. |
| Body-oriented effects and somatic experiencing | From my personal experience now, the empowering aspect lies in being able to trust the body and its ability to regulate itself. |
| Self- and resource-strengthening | For people/patients who have great diﬃculty establishing contact with themselves - for example, those who experience strong self-hatred or feelings of inner emptiness. Especially also for people who struggle to feel self-compassion or who experience strong guilt. |
| Exposure | Confrontation exercises through distancing from one’s own beliefs could be facilitated, especially in anxiety and obsessive–compulsive disorders. |
| Trauma-oriented effects | I could also imagine that it could help to process trauma by dealing with the experience during and after breathwork, accompanied by psychotherapy. |
| Risks |  |
| Psychological destabilization and symptom exacerbation | Defense mechanisms may be suspended; the patient may not yet be ready for the insights that arise during the session. This could potentially lead to overwhelm and a worsening of symptoms. |
| Risk to the therapeutic setting and patient boundaries | Abuse of power due to increased vulnerability of patients; metaphysical manipulation in the interpretation of extraordinary experiences; incompatible role expectations when acting as both therapist and facilitator. |
| Lack of support and insufficient after-care | I see risks more in the practical framework conditions of psychotherapeutic work—for example, not being able to provide suﬃcient space for integration due to capacity limitations, especially in more complex disorders. |
| Risk of dissociation | PTSD and dissociative disorders: flashbacks or dissociation may be triggered by the method. |
| Addictive potential | Dissociation, risk of psychosis, potential for addiction (?) |
| Spiritual bypassing | “spiritual bypassing”—experiencing profound emotions/insights that are not integrated into everyday life |
| No perceived risks | My assessment of relatively low risks is based on the assumption of responsible use within serious therapy, taking indications/contraindications into account, with trans-parent information, and within a stable therapeutic working relationship |

**Competing interests**

The authors declare no competing financial or non-financial interests.
